# Supplementary material for: Study on intestinal microbial communities of three different cattle populations on Qinghai-Tibet Plateau
Source: PLoS One. 2025 Feb 4;20(2):e0312314. doi: 10.1371/journal.pone.0312314 (PMC11793767; doi:10.1371/journal.pone.0312314)
Supplement: Attachment — Submitted filename: Response to Reviewers.docx [file pone.0312314.s002.docx]

**Response letter**

**(****Manuscript ID: PONE-D-24-36513R1)**

**Dear *PLOS ONE* Editor and Reviewer,**

Thank for your review comments on our manuscript entitled “Study on intestinal microbial communities of three different cattle populations on Qinghai-Tibet Plateau” (Manuscript ID: PONE-D-24-36513R1). These comments are all valuable and very helpful for improving our manuscript. We have made the corrections. We are hoping that this will meet the approval criteria. We use a change tracking mode to show what needs to be changed the main corrections in the manuscript and the responses to comments are as follows.

**Editorial modification**

We've checked your submission and before we can proceed, we need you to address the following issues:

Question 1

Thank you for stating the following financial disclosure: “This work was supported by the demonstration and promotion of healthy cattle breeding technology in Ganggu Village (SD2019XM008); and the project of genomic hypoxic selection signal and gene regulation network of continuous altitude cattle populations (32260823).”

Please state what role the funders took in the study. If the funders had no role, please state: "The funders had no role in study design, data collection and analysis, decision to publish, or preparation of the manuscript." If this statement is not correct you must amend it as needed. Please include this amended Role of Funder statement in your cover letter; we will change the online submission form on your behalf.

Answer 1

Thanks for your valuable comments, we have added a note to the funders in the "Additional Information" document that "funders have no role in the study design, data collection, analysis, publication decisions or manuscript preparation".

Question 2

PLOS requires an ORCID iD for the corresponding author in Editorial Manager on papers submitted after December 6th, 2016. Please ensure that you have an ORCID iD and that it is validated in Editorial Manager. To do this, go to ‘Update my Information’ (in the upper left-hand corner of the main menu), and click on the Fetch/Validate link next to the ORCID field. This will take you to the ORCID site and allow you to create a new ID or authenticate a pre-existing ID in Editorial Manager.

Answer 2

According to your suggestion, we have updated the personal information

Question 3

Please amend the title either on the online submission form or in your so that they are identical.

Answer 3

Thanks to your valuable comments, we have made consistent changes to the topic

**Reviewer 1**

**Comments to the Author:**

In the: Study on intestinal microbial communities of three different cattle populations on Qinghai-Tibet Plateau”following are some recommendations.

Question 1

Abstract:The resoult (linee 16) should be spell checked. Revise whole manuscript for such type of mistakes. Introduction:local species developing a strong adaptability to their environment. Despite the harsh environment, The Tibetan Plateau (The should be the). The practical applications of study are missing. The data is not fully supported by recent literature.

Answer 1

Thanks to the reviewer's valuable comments, we have made modifications. we have corrected the spelling throughout the entire article. Furthermore, we have incorporated recent literature and additional descriptions to enhance our discussion on the adaptability of native species from the Tibetan Plateau to high-altitude environments. The following references have been added: [3], [4], [5], [8], [9], [10], [17], [31], [33], [38], [40], [46], [48], [51], [55], [61], [62], and [63]. Specific content related to these references can be found in the manuscript at the following locations: lines 38-41, line 197, line 205, lines 222-223, line 230, line 231, line 248, line 259, line 267, and line.

Question 2

1. Materials and Methods: Experimental design can be represented in tabulated or pictorial form; Sample collection and transportation procedures are not justified.

Answer 2

Thanks for your comments, we have made modifications according to the requirements to further improve the information of the samples, and we have shown the experimental design in the form of pictures, as shown in fig. 1.

Question 3

2. Results: Scientific nomenclature (e.g. use of italics) should be followed throughout the manuscript. Check the manuscript for this carefully.

Answer 3

Thanks to the reviewer's valuable comments, we have made modifications. At the same time, we carefully checked the full text and italicized the names of the level microorganisms in the manuscript, namely *Coriobacterium* (lines 22 and 174), *Eubacterium*_*coprostanoligenes* (lines 165 and 167), *Phascolarctobacterium* (lines 167, 245-246, and 248), *Gastranaerophilales* (line167 and 245) and *Monoglobus* (line 167).

Question 4

Discussion: The latest Literature should be followed if possible. The discussion doesn’t seem to properly justify the results. There seems to be a lack of in-depth discussion. This portion needs to berevised. some more opposing references can also be added to strengthen this portion.

Answer 4

Thanks to the reviewer's valuable comments, we have made modifications. we have corrected the spelling throughout the entire article. we have incorporated recent literature and additional descriptions to enhance our discussion on the adaptability of native species from the Tibetan Plateau to high-altitude environments. The following references have been added: [31], [33], [38], [40], [46], [48], [51], [55], [61], [62], and [63]. Specific content related to these references can be found in the manuscript at the following locations: lines 38-41, line 197, line 205, lines 222-223, line 230, line 231, line 248, line 259, line 267.

**Reviewer 2**

**Comments to the Author:**

The manuscript " Study on intestinal microbial communities of three different cattle populations on Qinghai-Tibet Plateau " talks about the study of the intestinal microbial communities of cattle. The authors studied 3 different cattle population including one native and two hybrid populations. The two hybrid populations include Tibetan local cattle × Holstein cattle, TH and Tibetan local cattle × Jersey cattle, TJ and the native population is BL. They found that at the phylum level, the main microbial groups in the gut of these cattlepopulations were Bacteroidetes and Firmicutes. The authors did find several differences in the biomarkers of different microorganisms between the 3 cattle population. They found that Bacteroidales_RF16, Coriobacterium, and Muribaculaceae are associated with the immune system of BL cattle. The biomarkers in the intestines of TH cattle were mainly Oscillospiraceae and Clostridia UCG_014, while the biomarkers in the intestines of TJ cattle were Christensenellaceae and Gammaproteobacteria. This study is important because cross breeding of cattle can lead to better quality of cattle which are able to survive in native enviroment. This study suggested that BL cattle and TH cattle exhibit superior adaptability compared to TJ cattle, and that intestinal flora of cattle with different altitudes and breeds had different structures and functions. The manuscript is well written but I have a few concerns.

Question 1

The manuscript lacks figure legends and without thefigure legends it is difficult to interpret the results. Authors need to provide the figure legends and label the figures completely.

Answer 1

Thank the reviewers for their valuable comments, and we are very sorry for the mistakes caused by our carelessness. We have modified the drawing notes and legend of the manuscript, as detailed in the manuscript.

Question 2

The result section needs better headings, the heading are quite similar and at times confusing.

Answer 2

We would like to express our gratitude to the reviewers for their valuable comments. In response to their valuable feedback, we have implemented the following implemented: The title "3.2 Analysis of microbial diversity of three high-altitude cattle populations" has been revised to "Diversity changes of three high-altitude cattle populations microbial diversity "(line 143), Replace "3.3 Analysis of differences in the microbial composition of three high-altitude cattle populations" with "3.3 Analysis of differences in the gut microbial composition(line 156) ", Replace "3.4 Screening of microbial biomarkers from three high-altitude cattle populations" with "3.4 Screening of microbial. biomarkers from three cattle populations "(line 170). Replace "3.5 Prediction of microbial function in three high-altitude cattle populations" with "3.5 Functional predictions of the rectal microbiota in cattle using PICRUSt2 "(line 173).

Question 3

The difference in the intestine microbial population at the phylum level is very less for the abundant phyla. The authors should clearly state this as the finding is buried in the text. Firmicutes is over 76% (BL = 76.71, TH= 77.85, TJ = 78.44%) and abundance of Bacteroidetes was over 16% (BL =16.93%, TH = 16.81%, TJ = 16.44%).

Answer 3

Thanks for the reviewer's valuable comments, we have supplemented and elaborated on our manuscript in accordance with these insights. The reviewer noted, "Interestingly, at the phylum level, there was no significant difference between Bacteroides and Firmicutes in the gut microbiota across the three different elevations. This may be attributed to the uniform feeding of the same Total Mixed Ration (TMR) sourced from the same company, which likely results in minimal variations in the dominant phyla of the microbiotas among the populations (lines 242-245).

Question 4

Minor Concern: Lin 253 has an error and Figure 2 needs better labellineg .

Answer 4

Thank the reviewers for their valuable comments. According to the reviewer's valuable comments, we have made modifications and added tags to Figure 3 (formerly Figure 2). For further details, please refer to the manuscript.

We hope this revised manuscript has addressed your concerns, and look forward to hearing from you.

Yours sincerely,

Quji Suolang.

Institute of Animal Husbandry and Veterinary Science, Tibet Academy of Agricultural and Animal Husbandry Sciences

E-mail:547692283@qq.com
